# Impact of the COVID-19 Pandemic on the Practice of Hand and Upper Extremity Surgeons

**DOI:** 10.7759/cureus.12072

**Published:** 2020-12-14

**Authors:** Uzair A Qazi, Julianne Sutton, Scott C Farner, Laxminarayan Bhandari

**Affiliations:** 1 Hand Surgery, Kleinert Kutz and Associates for Hand and Microsurgery, Louisville, USA; 2 Research, Kleinert Kutz and Associates for Hand and Microsurgery, Louisville, USA

**Keywords:** covid 19, hand surgery

## Abstract

Objective

The purpose of this study was to determine the impact of the coronavirus disease 2019 (COVID-19) pandemic on the practice of hand and upper extremity surgeons.

Methods

We assessed how the pandemic affected the practice on multiple fronts including professional, personal, and practice aspects. The survey was conducted through an online questionnaire that had six sections: demographics, clinic, elective surgery, emergency surgery, urgent surgery, and human resources. The survey was sent to 586 Kleinert Society members who are all practicing hand and upper extremity surgeons.

Results

We received 35 responses from the United States and 53 from the rest of the world. Based on our findings, the clinic volume was reduced by >50% in the early stages, subsequently returning to a level that was 25-50% lower than pre-COVID-19 times in later stages. A corresponding decrease in elective surgeries was also noted. The need for preoperative COVID-19 tests added to the logistics of surgery, causing delays of three to six hours for emergency cases and >24 hours for urgent cases. The hand surgeons witnessed multiple furloughs, layoffs, and even COVID-19 infections among nursing and support staff. Most hand surgeons continued to perform urgent and emergency surgeries during the pandemic. The application of telemedicine was not popular and had multiple drawbacks. Hand surgeons are modifying their practice by adopting measures such as social distancing, reducing the clinic volume, and using personal protective equipment (PPE).

Conclusions

As COVID-19 is likely to prevail for the foreseeable future, these measures are here to stay. The initial reduction in the clinic and elective volume has improved but has not reached pre-COVID-19 levels, suggesting a slow recovery. As reopening measures will lead to more people rejoining employment, subsequently, more patients with hand-related conditions are likely to present to the clinics. Rapid COVID-19 testing and supply of PPEs will play a crucial role in the near future to enable hand surgeons to continue their service while taking care of their personal health.

## Introduction

The coronavirus disease 2019 (COVID-19) outbreak has assumed pandemic status, causing many governments to impose lockdown measures to control the spread. As the medical systems worldwide geared up to face the pandemic, resources were reallocated to meet the needs of COVID-19 care. Clinics and elective surgeries were reduced or suspended. Furloughs, layoffs, and reassignments affected the clinic staff along with sickness caused by the disease itself. Furthermore, the lack of personal protective equipment (PPE) and uncertainty regarding precautionary measures further strained the medical practice. Gradually, governments moved to reopening or unlocking measures. Clinics have restarted along with offering elective surgeries in some cases. Telemedicine, social distancing, COVID-19 testing, and PPE have become the new normal.

The pandemic has affected every specialty to a different degree. It would be interesting to note how the hand surgeons have been affected by the current pandemic, both during the lockdown and reopening phases. Hand surgery entails a wide variety of emergency, urgent, and elective cases. It is unique in that a large portion of the surgery lies in the grey area of emergent and non-emergent surgery. A comprehensive analysis of the effect of COVID-19 on hand surgery has not been attempted so far.

The purpose of this study was to determine the impact of the current COVID-19 pandemic on the practice of hand and upper extremity surgeons on multiple fronts including professional, personal, and practice aspects. The data accumulated through this questionnaire will give an insight into how COVID-19 affected hand surgeons during the lockdown phase and also during the reopening phase. An understanding of the various challenges faced and how they were addressed will be extremely helpful to hand surgeons worldwide. Furthermore, this data can form the basis for guidelines on how to safely continue providing care during the pandemic.

## Materials and methods

In order to understand the effect of COVID-19 on hand and upper extremity surgeons, a questionnaire was formulated. The questionnaire had six sections: demographics, clinic, elective surgery, emergency surgery, urgent surgery, and human resources. The Stanford guidelines for elective, urgent, and emergency surgery were selected, which are as follows: (a) elective surgery: treatment is necessary but can be delayed for at least 30 days. Delay of surgery may be remediated by medical management. (b) urgent surgery: necessary due to a threat of losing life, limb, organ, or a permanent disability and/or necessary for the progression of treatment for life, limb, organ disability within 30 days. Urgent is also defined as follows: in the physician's judgment, delay of the operation or procedure would cause harm to the patient or in delayed diagnosis. (c) emergency: necessary due to the immediate threat of losing life, limb, organ, or a permanent disability [1].

The questionnaire was sent out using an online service (SurveyMonkey, San Mateo, CA). The survey was sent to members of the Kleinert Society, which constitutes hand surgeons who had their fellowship training at the Christine M Kleinert Institute of Hand and Microsurgery, Louisville, KY. The rationale for selecting the Kleinert Society list was that the cohort consisted predominantly of hand surgeons in the United States while simultaneously including a world-wide distribution of hand surgeons. The data were analyzed by the Wilcoxon test for paired data as well as descriptive statistics using RStudio version 3.6.6.

## Results

**Figure 1:**
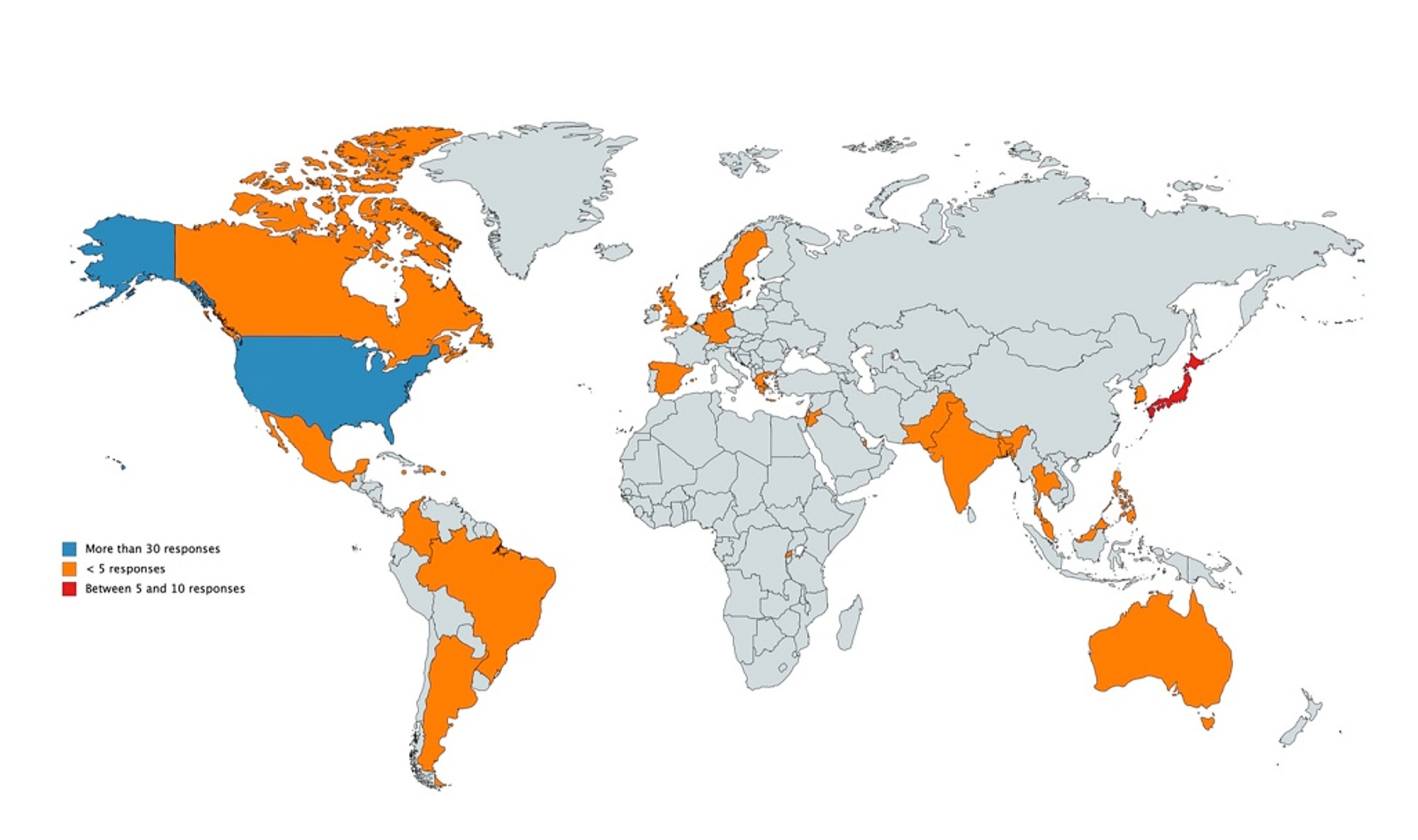
Map representation of responses from various countries

Out of a total of 586 emails that were sent, 37 (6.3%) bounced and seven (1.2%) recipients opted out of the survey. Eighty-eight responses were received. The response rate of 15.01% was comparable to that of other similar-sized surveys [2]. The respondents belonged to different countries, as shown in Figure 1.

Section 1: demographics

**Figure 2:**
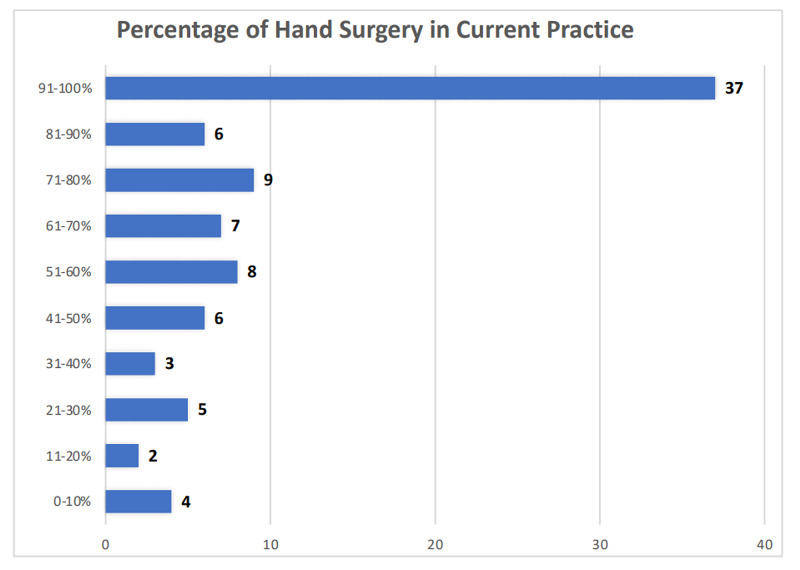
Percentage of hand surgery in current practice The chart shows the percentage of hand surgery in respondents' clinical practice

The majority of the respondents reported that hand surgery constituted >90% of their practice (Figure 2).

**Figure 3:**
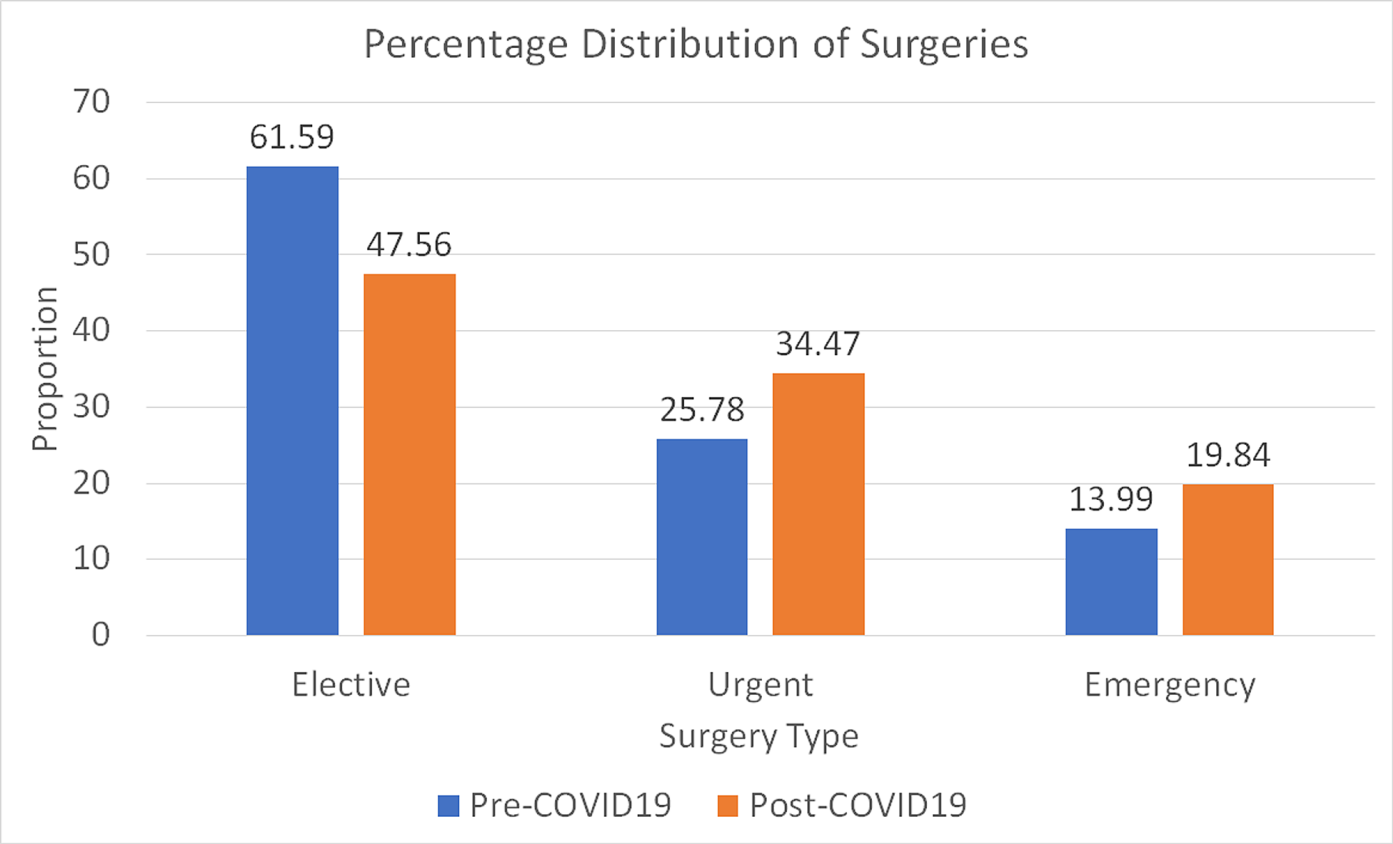
Percentage distribution of surgeries The chart shows the percentage distribution of elective, urgent, and emergency surgeries. The blue bar represents pre-COVID-19 proportions, whereas the orange bar represents post-COVID-19 onset proportions COVID-19: coronavirus disease 2019

The Wilcoxon test for paired data was used to evaluate the change in proportions of elective, urgent, and emergency surgeries before COVID-19 and during COVID-19. The reduction in elective cases was found to be significant (p-value: <.001), Similarly, a significant increase in urgent and emergency surgeries were noted (p-value: <.001) (Figure 3) [3].

**Figure 4:**
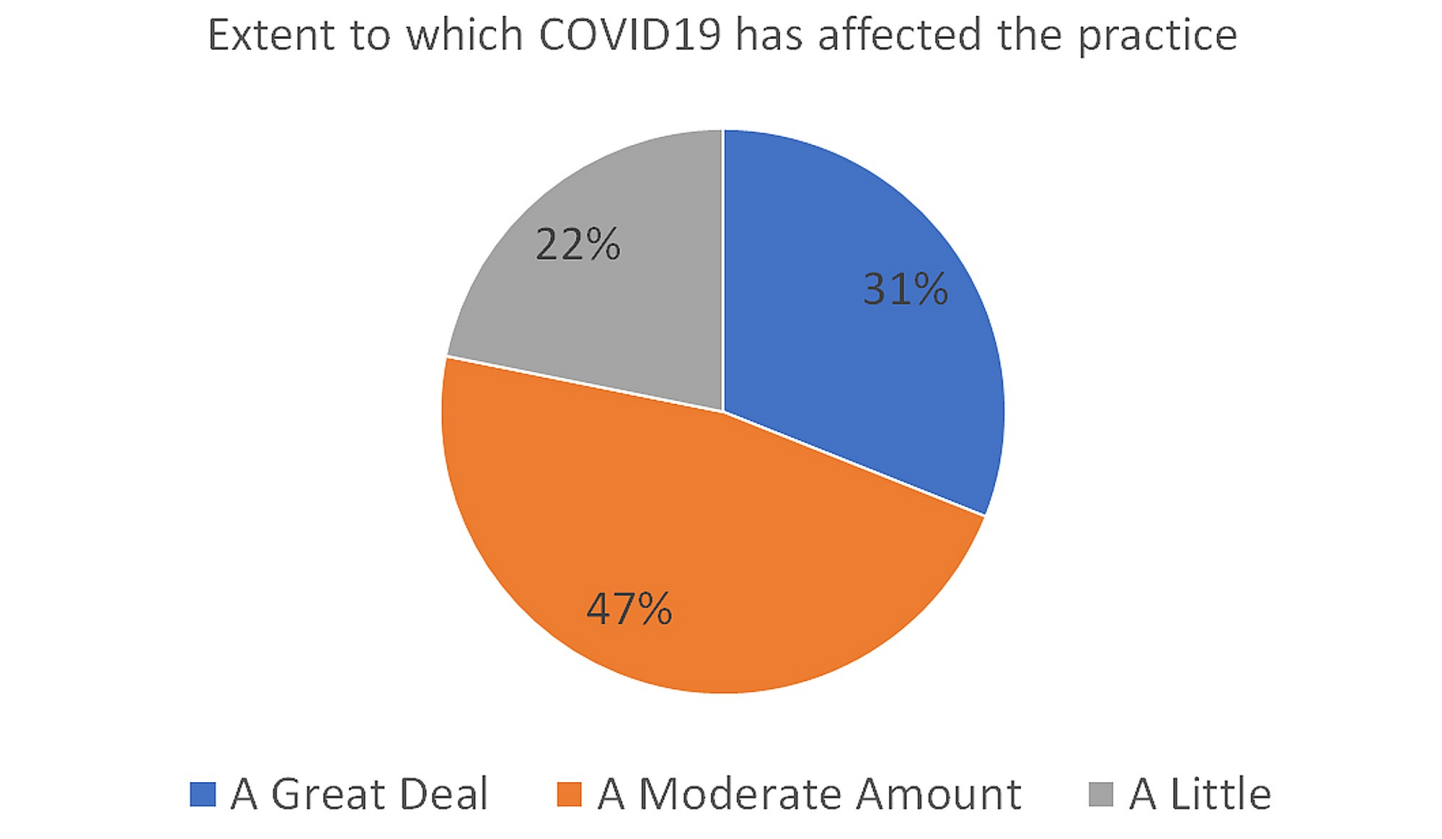
Extent to which COVID-19 has affected the practice The chart shows the overall extent to which the COVID-19 pandemic affected the practice of hand surgeons COVID-19: coronavirus disease 2019

When asked about how the COVID-19 pandemic had affected their practice, 31% responded that the pandemic had affected their practice a great deal, 47% reported that it was affected moderately, and 22% said that they were only a little bit affected (Figure 4).

Section 2: clinic

**Figure 5:**
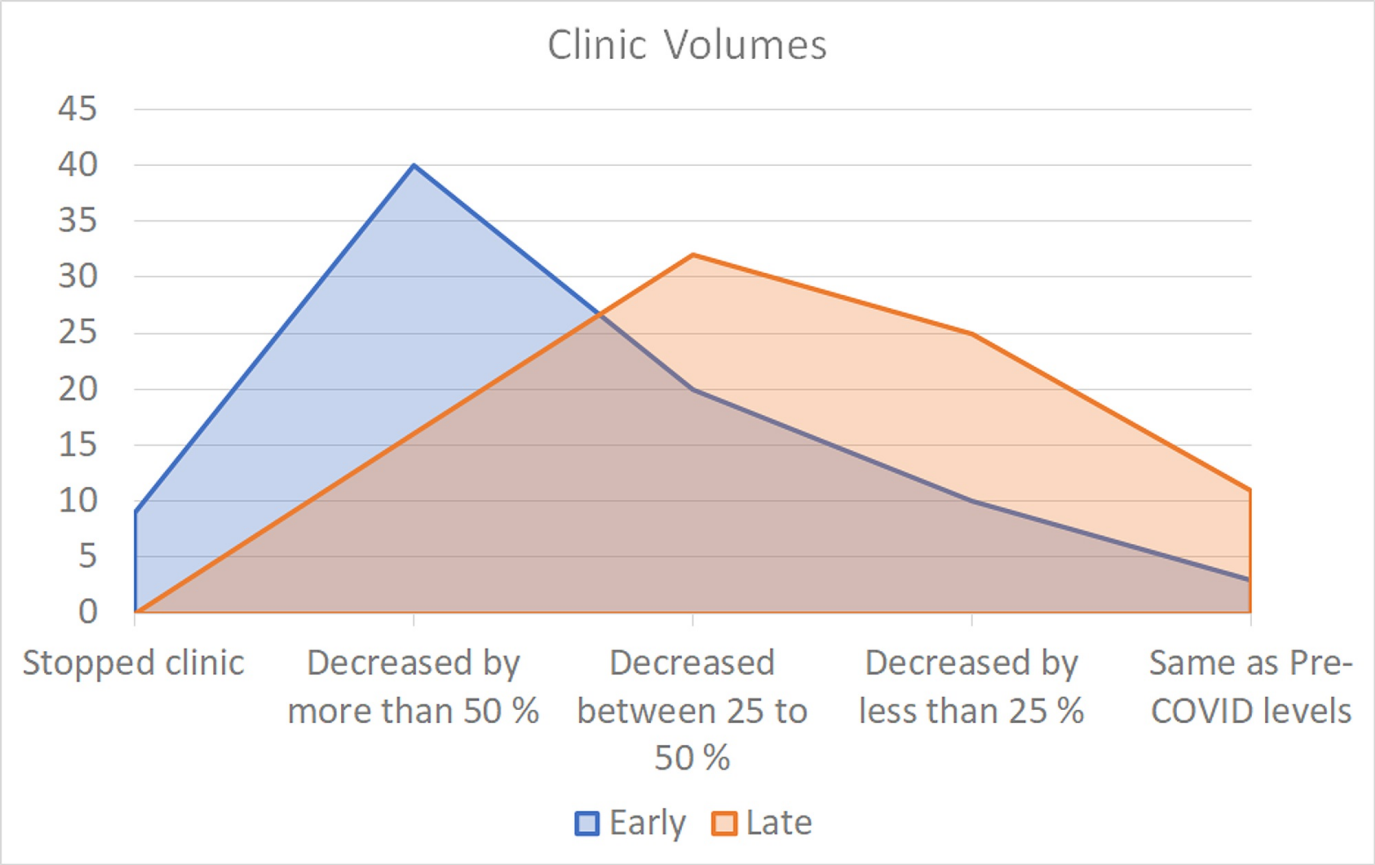
Clinic volumes The chart shows a comparative analysis of how hand surgeons around the world were affected by COVID-19 during the early and late periods of lockdown COVID-19: coronavirus disease 2019

This section focused on the impact of the pandemic on clinic visits. For 47.06% of respondents, the clinic volume decreased by more than half. For 23.53% of respondents, the clinic volume decreased by 25-50%. During the late period, the clinic volumes started to improve, and only 18.82% of the participants found their clinic volume to be lower than 50%. For 37.65%, their practice was back to 25-50% of its pre-COVID-19 volume (Figure 5).

**Figure 6:**
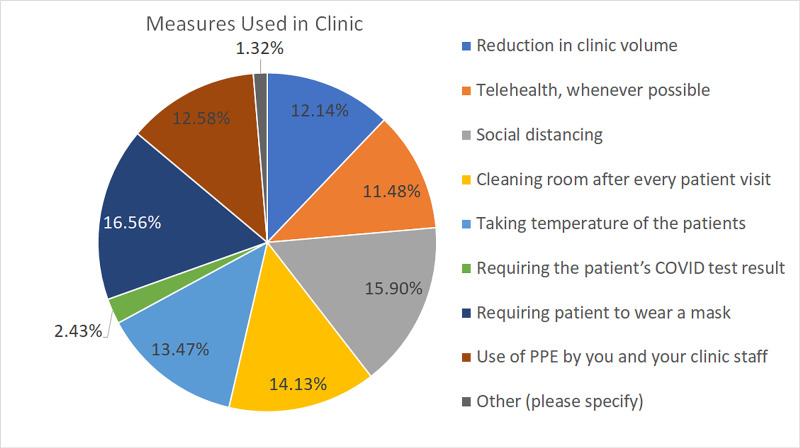
Measures used in clinics The chart shows the measures used in clinics as part of mitigation protocols during the COVID-19 pandemic COVID-19: coronavirus disease 2019; PPE: personal protective equipment

Several measures were taken to mitigate the spread of COVID-19, and the most notable among them are depicted in Figure 6.

**Figure 7:**
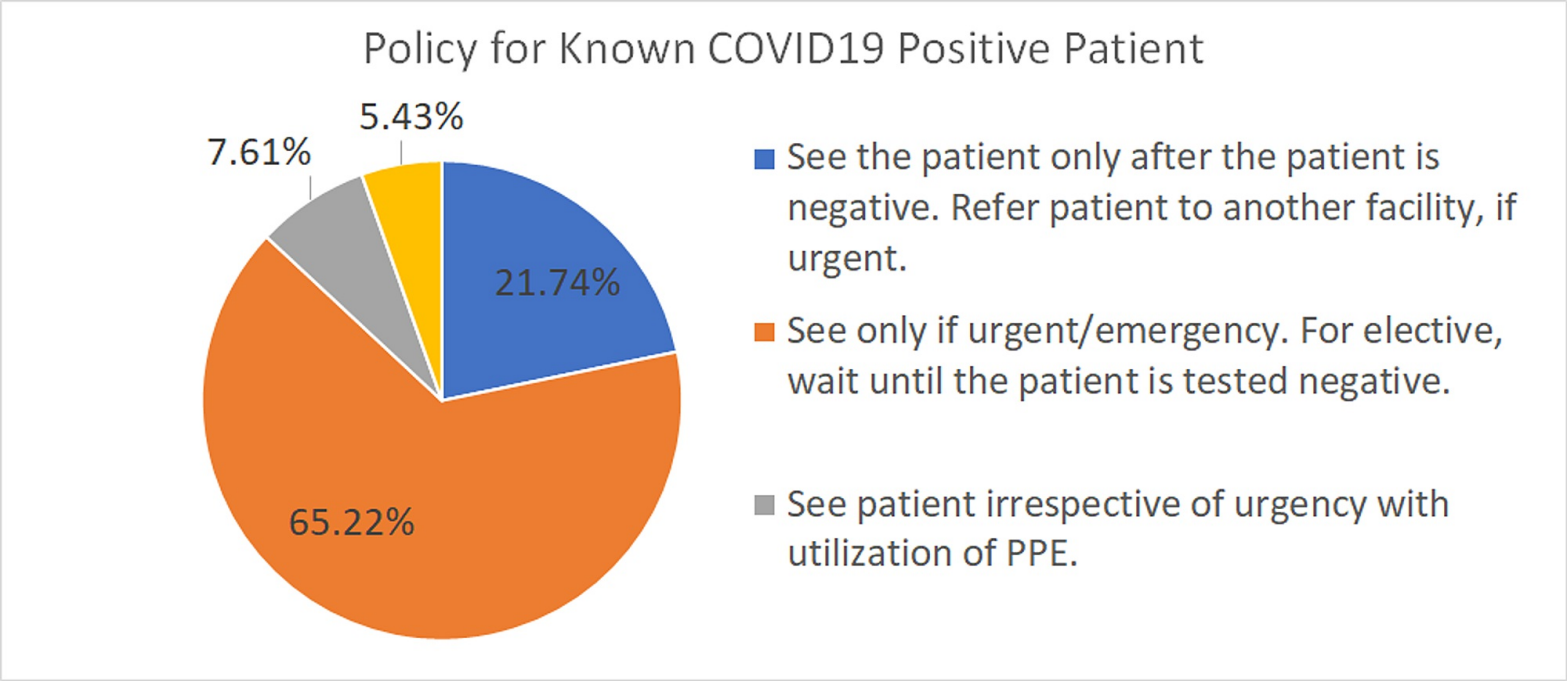
Policy for a known COVID-19-positive patient The chart shows the policies used for a known COVID-19-positive patient COVID-19: coronavirus disease 2019; PPE: personal protective equipment

A known COVID-19-positive patient was seen only for an urgent/emergency indication by 65.22% of our respondents, and they waited for the patient to test negative if it was an elective case. Whereas, 7.61% saw the patient donning PPE (Figure 7).

**Figure 8:**
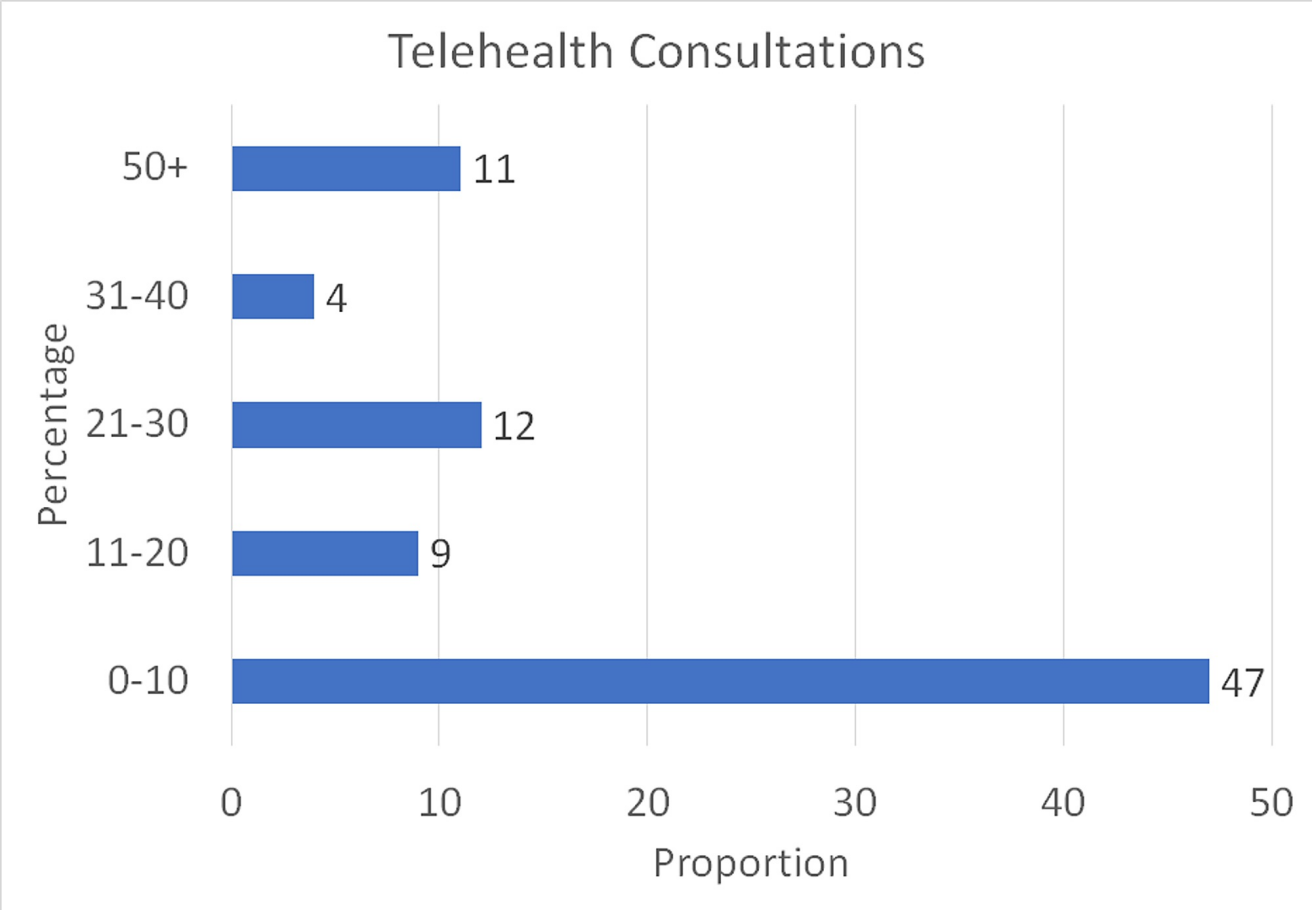
Telehealth consultations The chart shows the extent to which telehealth consultations were used in the hand surgery practices of our respondents

Only 11.48% of our respondents used telemedicine for their clinic visits, and among them, the majority of the respondents (56.6%) used it only for <10% of patients (Figure 8).

**Figure 9:**
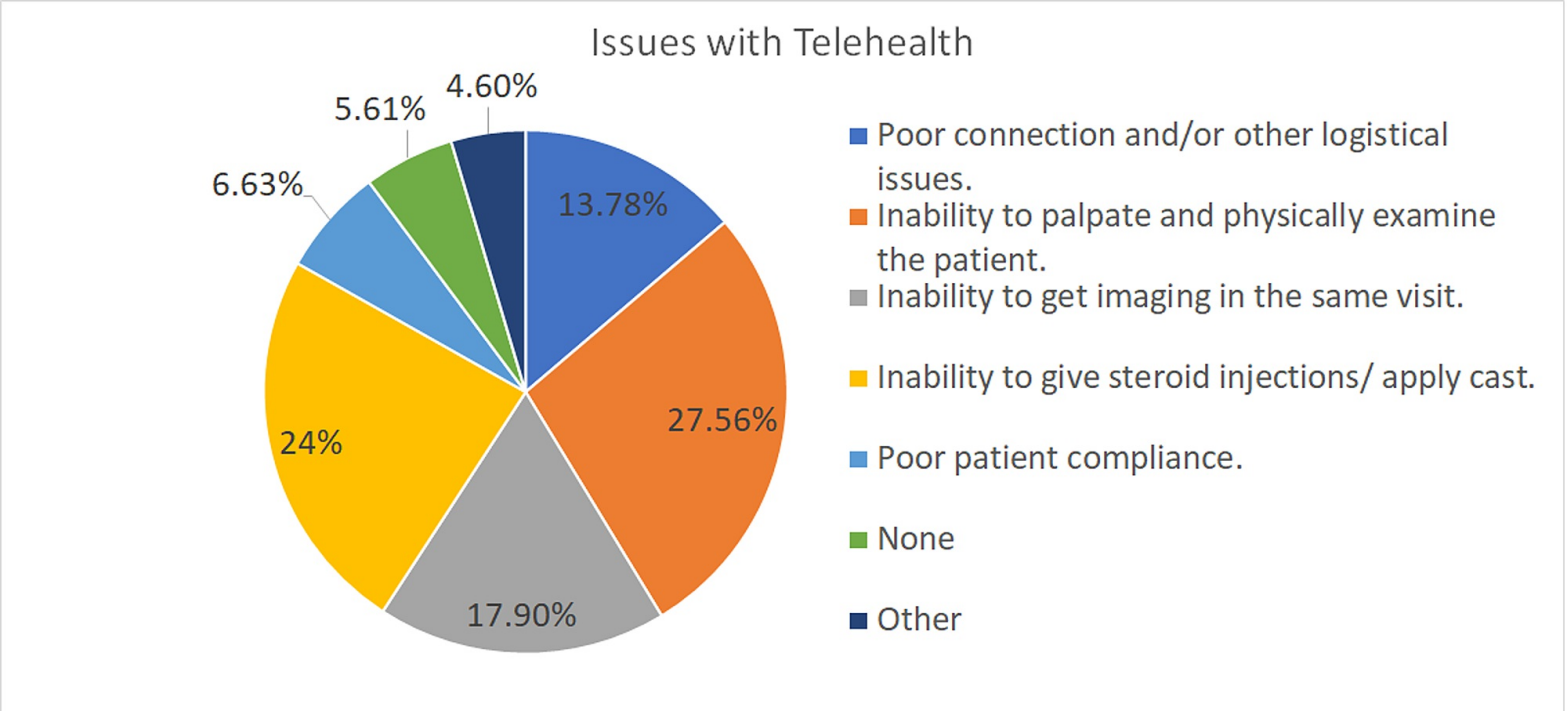
Issues with telehealth The chart shows the issues experienced by the respondents when using telehealth

Various drawbacks of telemedicine in hand surgery were cited by the responders, which are listed in Figure 9.

Sections 3-5: surgeries

The surgeries were divided into emergency, urgent, and elective based on the Stanford guidelines [1]. The third section of the questionnaire focused on emergency hand surgeries while the fourth section assessed urgent hand surgeries, and the fifth was for elective surgeries.

**Figure 10:**
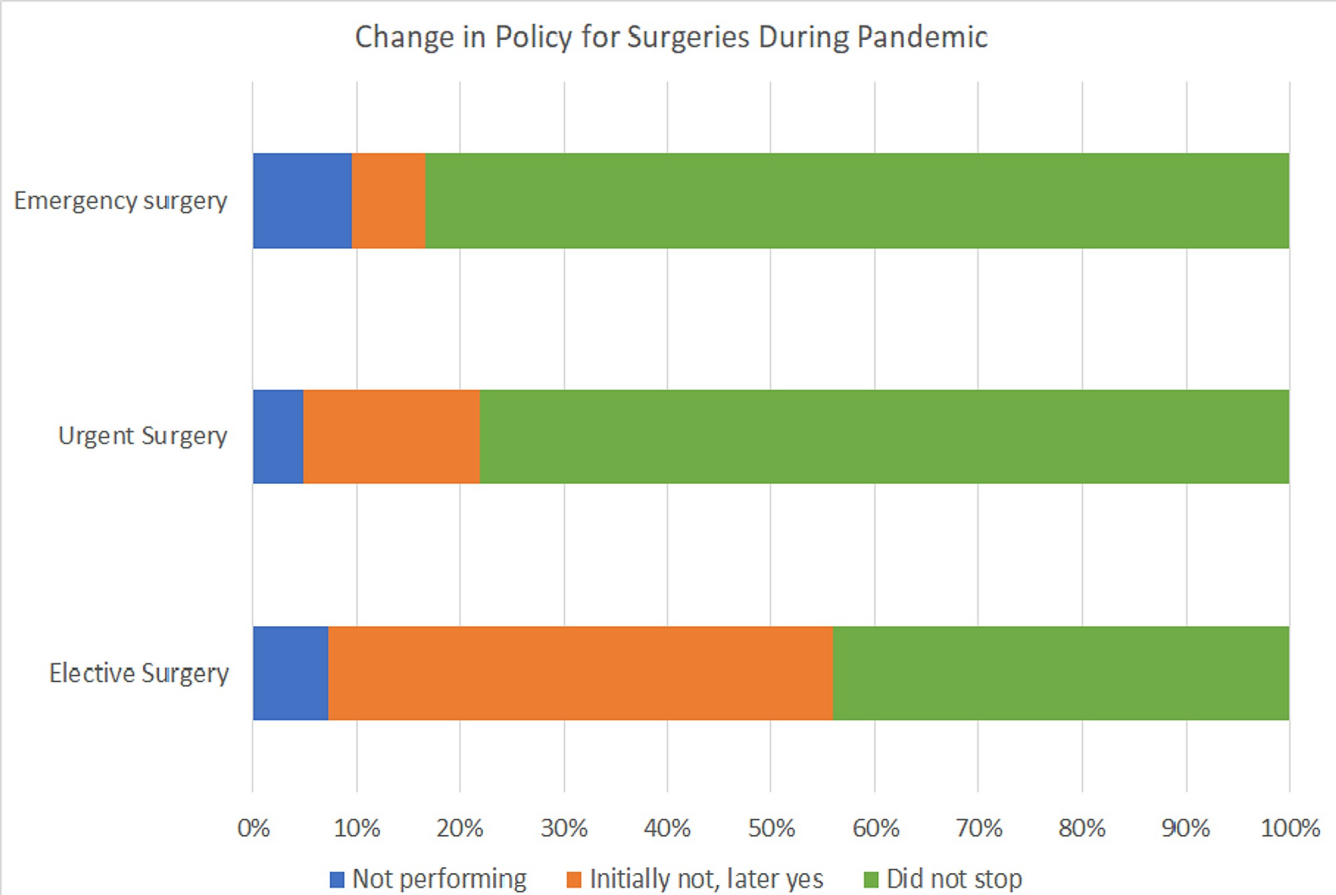
Change in policy for surgeries during the pandemic The chart shows the change in policy for elective, urgent, and emergency surgeries during the pandemic

Elective surgeries were stopped completely by 56.1% of the respondents in the early period and 7.32% of the respondents in the late period. Emergency surgeries were never stopped by 83.33% of respondents, while 78% never stopped urgent surgeries during the pandemic (Figure 10).

**Figure 11:**
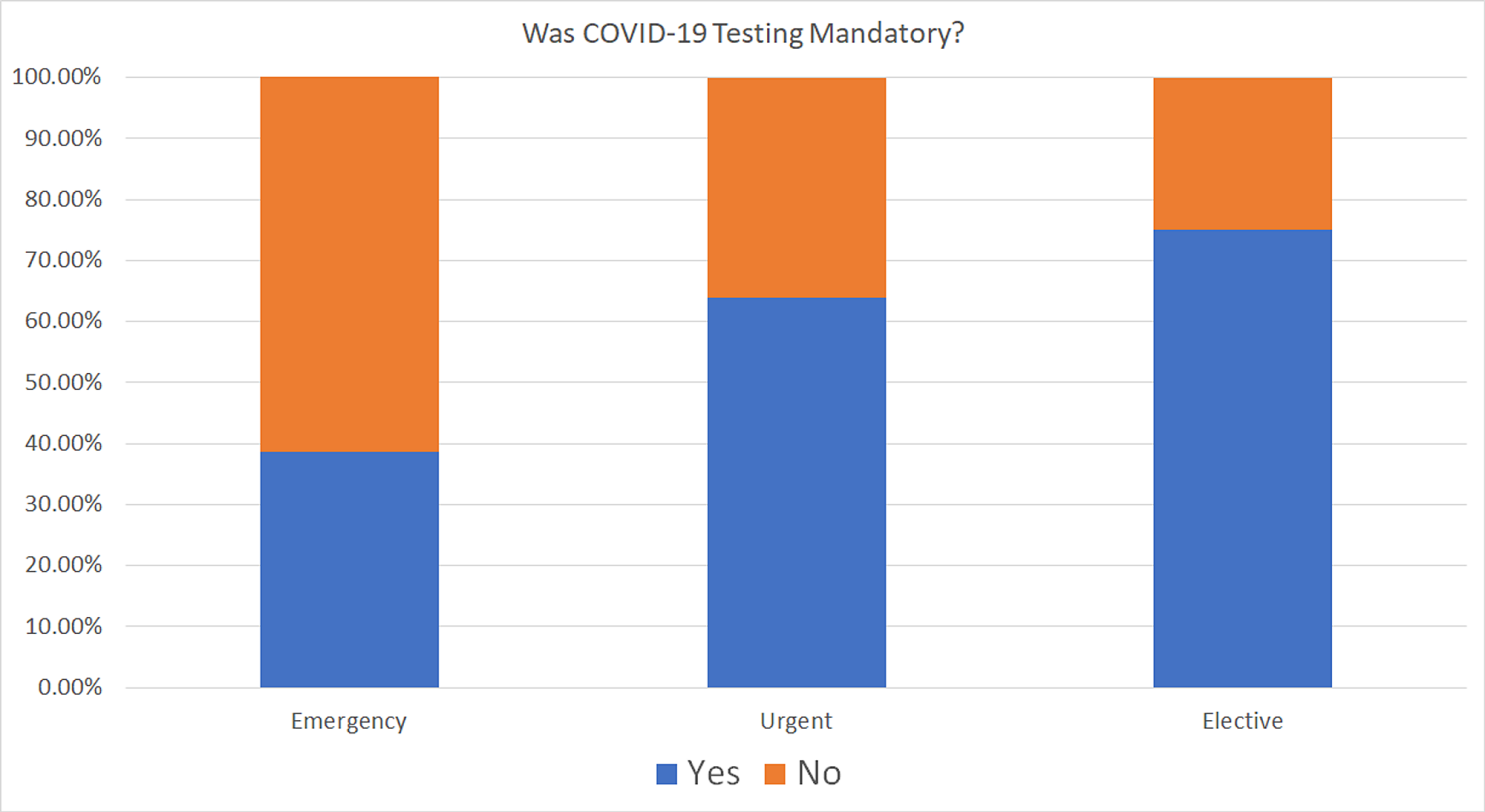
Mandatory COVID-19 testing The chart shows if COVID-19 testing was mandatory for emergency, elective, and urgent cases COVID-19: coronavirus disease 2019

While 38.55% of respondents reported a mandatory requirement for COVID-19 testing for their emergency patients, the numbers increased to 63.86% for urgent cases and 75% for elective cases (Figure 11).

**Figure 12:**
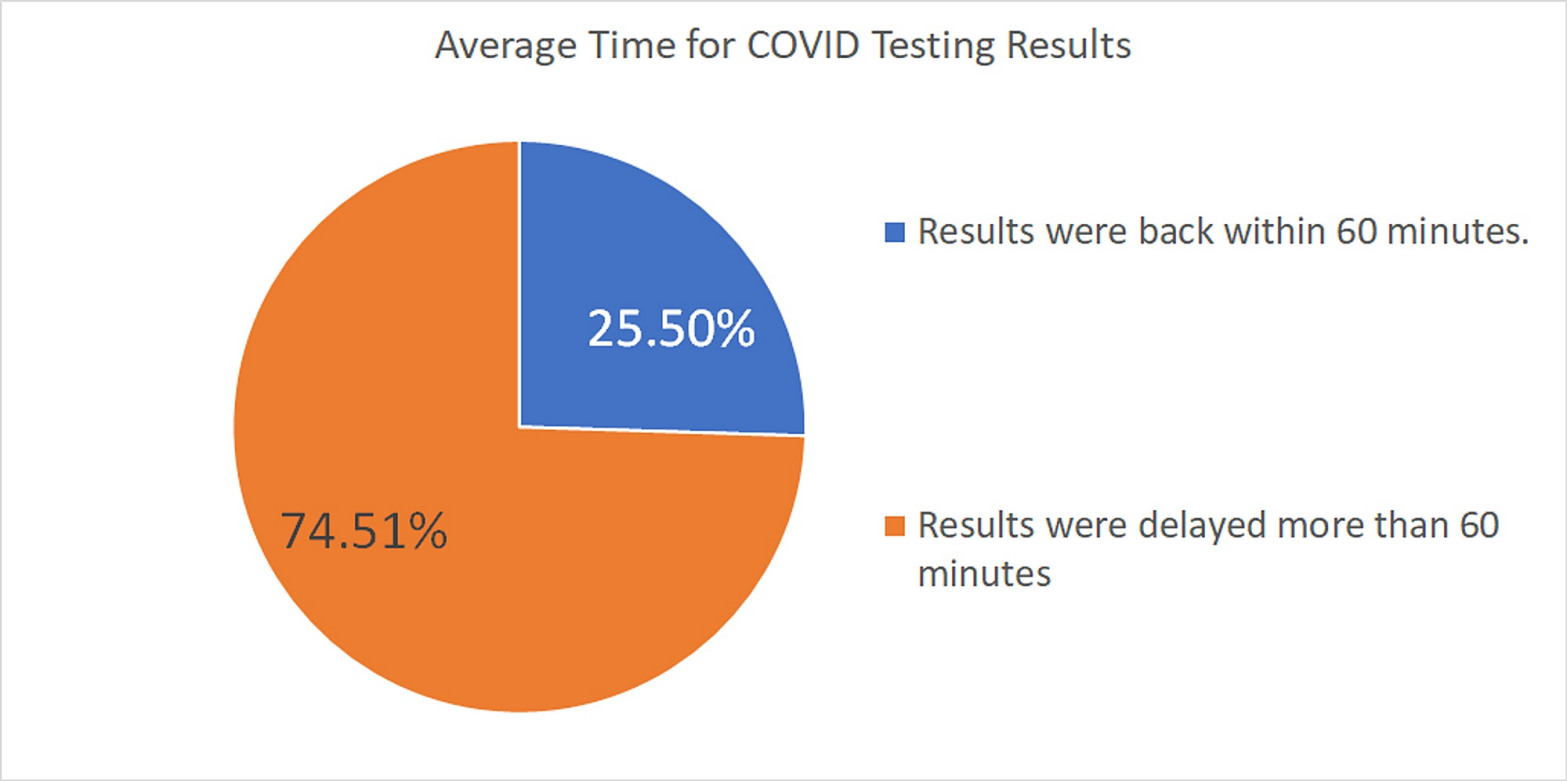
Average time for COVID-19 testing results The chart shows the percentage of COVID-19 test results coming back within 60 minutes and those that took longer than 60 minutes COVID-19: coronavirus disease 2019

The time it took to get the COVID-19 testing results back was also assessed. For an emergency situation, 74.51% of respondents reported a delay of over one hour (Figure 12).

**Figure 13:**
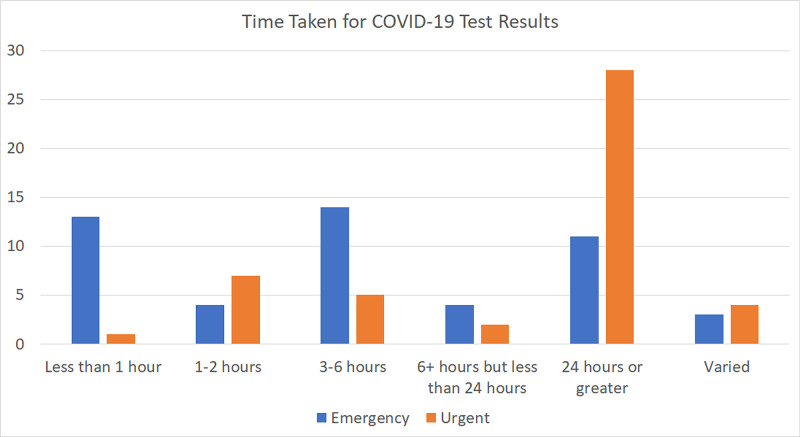
Time taken for COVID-19 test results The chart shows the average delay for COVID-19 test results to come back in emergency and urgent cases COVID-19: coronavirus disease 2019

A breakdown of the time taken for test results to come back for emergency and urgent cases is shown in Figure 13.

As the time taken was not a significant factor for elective cases, it was not assessed.

**Figure 14:**
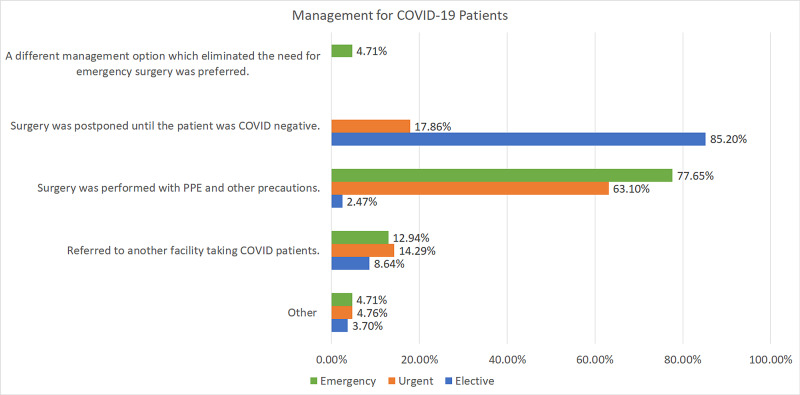
Management of COVID-19 patients The chart shows the policy for COVID-19-positive patients regarding elective, urgent, and emergency surgeries COVID-19: coronavirus disease 2019

If a patient was found to be COVID-19-positive prior to emergency surgery, 77% responded that they performed the surgery with PPE and other precautions, whereas 13% preferred to refer the patient to a facility that was taking COVID-19 patients. For urgent surgeries, if a patient tested positive for COVID-19, 63% would still perform it using PPE and other mitigation protocols, whereas 18% would postpone it until the patient was tested negative for COVID-19. However, for elective surgery, 85% of respondents opted to postpone the surgery, whereas 9% referred the patient to a facility accepting COVID-19-positive patients, and only 2% performed surgery with PPE (Figure 14).

Section 6: human resources

The last section of the survey focused on the effects of the pandemic on our respondents and their employees. Layoffs were noted by 51% of respondents in their practice, and 10% reported being laid off themselves. Regarding COVID-19 infection among healthcare workers, 57% reported their staff being diagnosed with COVID-19, while 2% of respondents had COVID-19 themselves; 83% of the respondents said that their workplace did not require them to test for COVID-19.

## Discussion

The World Health Organization declared the rapid spread of the severe acute respiratory syndrome coronavirus 2 (SARS-CoV-2) virus a pandemic on March 11, 2020 [4]. The total number of cases has crossed 30 million and has resulted in over 900,000 deaths worldwide [5]. Like many other specialties, hand surgery has also been affected by the reduced anesthesia support, reduced number of beds for inpatient stays, and the reassignment of hand surgery staff and doctors to pandemic-related duties, etc. [6]. Many medical and surgical societies got together and devised various strategies on how to balance the surgical and safety needs of the patients and, despite constraints, how to best manage their resource allocation and contribute to mitigation efforts in place during the pandemic [7-13].

The current survey was done to evaluate the professional and personal impact of COVID-19 on the practice of hand surgeons. The survey encompassed the impact in the early period of the pandemic, which was from January to April 2020, and the late period from May 2020 onwards when lockdown restrictions were being eased out.

According to our survey, the impact on hand surgery practices around the world was significant (Figure 4). Nearly half of the respondents noted >50% reduction in clinic volume, and 10% had stopped clinic at the early stages of the COVID-19 pandemic. While these numbers improved during the late stage, only 13% have reported it to reach the pre-COVID-19 levels, suggesting a long road back to normalcy. As lockdown measures are still being lifted, more and more people will go back to work, travel, and participating in recreational activities, which is likely to result in an increase in hand-related conditions presenting to clinics.

The reduction in clinic volume appears to have affected elective surgeries. The reduction in the percentage of elective surgeries and the corresponding increase in urgent and emergency surgeries were found to be statistically significant. The percentage increase in emergency and urgent cases is a relative one resulting from a reduction in elective cases. Therefore, the dynamics of a hand surgeon's practice has changed from predominantly elective cases to more urgent and emergency cases.

Hand surgeons adopted numerous strategies to restart clinics safely. The most common ones were mandatory mask usage (88.24%), mandatory social distancing (84.71%), reducing clinic volumes intentionally (64.71%) to avoid gathering of patients in the waiting area, and allowing time for room cleaning in between patients (75.29%). Other measures included not allowing patient bystanders, provision of adequate hand sanitizers, asking the patient to wait in their car until they are called in, and screening the patients on arrival. Another strategy was telehealth. However, only 11.48% of our respondents used telemedicine for their clinic visits, and among them, the majority of the respondents (56.6%) used it only for <10% of their patients. The lack of popularity of telemedicine in hand surgery is due to numerous reasons. These reasons include the inability to palpate and physically examine the patient (68.35%), inability to give steroid injections/apply casts (59.49%), being unable to get imaging done in the same visit (44.30%), and logistical or connectivity issues (34.18%).

As reopening measures were being adopted, patients were being tested preoperatively for COVID-19. Mandatory COVID-19 testing for their emergency patients was reported by 38.55% of respondents. The numbers increased to 63.86% for urgent cases and 75% for elective cases. The drawback of this requirement was the prolonged wait times; patients had to wait to get their surgeries done. For emergency cases, 74% of respondents reported a delay of over an hour. In most cases, the delay for emergency cases was three to six hours, and for urgent cases, it was more than 24 hours.

If a patient was found to be COVID-19-positive prior to emergency surgery, 77% of respondents performed surgery with PPE and other precautions, whereas 13% referred the patient to a facility that was taking COVID-19 patients. For urgent surgeries, 63% would still perform it using PPE and other precautions, whereas 18% would postpone it until the patient was tested negative for COVID-19. However, for elective surgery, 85% of respondents opted to postpone the surgery, whereas 9% referred the patient to a facility accepting COVID-19-positive patients, and only 2% performed surgery with PPE.

This brings into focus the benefit of COVID-19 screening for emergency cases. For centers equipped with handling COVID-19 patients, the only effective difference the COVID-19 tests makes is the usage of PPE and other precautions. If the supply of such materials is not an issue, then universal COVID-19 screening can be replaced with universal precautions. This not only saves time, which is critical in an emergent condition, but also reduces the burden on testing resources, which could be reserved for patients whose treatment depends on the diagnosis. We recommend a critical analysis by healthcare administrators to reassess universal COVID-19 testing for emergency and urgent cases.

The final section of the survey focused on issues related to human resources. While 10% of our respondents reported being laid off themselves, 51% had some of their staff laid off; 2% of respondents were diagnosed with COVID-19, while an astonishing 57% had their staff diagnosed with COVID-19. This highlights the high risk that surgeons and their staff put on themselves while offering their services during the pandemic.

Other researchers have studied the effect of the pandemic on many different specialties such as orthopedic surgery, maxillofacial surgery, plastic surgery, minimal access surgery, and transplant surgery [6-16]. However, a comprehensive analysis of the effect of COVID-19 on hand surgery has not been done. There were only two surveys that looked at some aspects of the pandemic on hand surgery. Ducournau et al. in a survey of 47 hand surgeons looked at how COVID-19 patients were handled by hand surgeons worldwide [16]. The authors suggested that an international consensus is needed for the management of COVID-19 patients. In order to reach a consensus, transnational data is needed, which is provided by our study. Our study looks not only at the clinical practice aspects but also at clinical volume and human resource issues. The second study on COVID-19 in the field of hand surgery was done by Hwee et al. [6]. They shared a single-center experience during the early period of COVID-19.

Our study assessed the overall impact of the pandemic on a wide scale. The pandemic has affected hand surgeons on multiple levels, and the current study showed how that happened. All aspects of hand surgery practice were covered, and this remains to this date the most comprehensive survey done. We received responses from 31 different countries and from all continents. All respondents were fellowship-trained hand surgeons with the majority having >90% of their practice focusing on hand surgery. The current study documented a significant reduction in elective cases and clinic volume, use of various mitigation measures, the way the pandemic altered the nature of the practice, staff layoffs, drawbacks of telemedicine, and delay in surgeries due to COVID-19 testing. As this pandemic is likely to persist in the foreseeable future, the hand surgeon has to adapt to these changing and testing times. This survey helps to picture a hand surgeons’ practice in the future. As telemedicine has significant drawbacks in hand surgery, physical visits would be necessary. Social distancing, universal masking, and frequent hand-washing would be the norm. For urgent and emergency cases, universal COVID-19 testing may be replaced with universal PPE measures. While the clinic volumes and elective surgeries continue to increase, the return to normalcy will likely take a considerable period of time. Meanwhile, hand surgeons and their staff continue to be at risk.

The current study is not without its drawbacks. All the drawbacks associated with a survey would apply to this case as well, including a recall bias and a desirability bias. There was no way to verify the responses. A higher response rate was desirable. The cohort was heterogeneous and diverse related to age, experience, and location of the practice. However, we believe this to be a strength as such a diverse sample is more likely to reflect reality.

Recommendations

Based on the findings of the current study, the following recommendations are made:

1. Adopting rapid tests for urgent and emergency cases. Alternatively, doing away with mandatory preoperative COVID-19 testing and adopting universal PPE for urgent and emergency cases can be considered.

2. Securing an uninterrupted supply of PPE so that surgeons are not affected by the delay caused by COVID-19 testing.

3. As the trend shows that clinic volumes are returning to normal levels, social distancing, masks, and other precautions should be universally applied.

4. Further assessments of the financial aspect of hand surgery practice should be conducted.

The level of evidence assigned to this study is IV.

## Conclusions

COVID-19 pandemic has affected hand surgeons worldwide on multiple fronts. Our survey participants reported that the clinical practice volume was reduced by >50% during the initial period but has recovered to 25-50% in the later periods. Telemedicine has not been popular due to multiple drawbacks. Elective surgeries have declined. However, the majority of the respondents continued to perform urgent and emergency surgeries. Preoperative COVID-19 testing was mandatory in most cases causing delays of three to six hours for emergency cases and >24 hours for urgent cases. In case the patient was COVID-19-positive, most respondents continued to operate with PPE and other measures. Of note, 51% of the respondents reported staff being laid off or furloughed; 57% reported their staff being diagnosed with COVID-19. Thus, COVID-19 has affected hand surgeons on both professional and personal fronts, and the road back to normalcy is a tedious one.
